# The impact of postoperative cognitive training on health‐related quality of life and cognitive failures in daily living after heart valve surgery: A randomized clinical trial

**DOI:** 10.1002/brb3.2915

**Published:** 2023-02-13

**Authors:** Marius Butz, Tibo Gerriets, Gebhard Sammer, Jasmin El‐Shazly, Marlene Tschernatsch, Patrick Schramm, Thorsten R. Doeppner, Tobias Braun, Andreas Boening, Thomas Mengden, Yeong‐Hoon Choi, Markus Schoenburg, Martin Juenemann

**Affiliations:** ^1^ Heart and Brain Research Group Kerckhoff Heart and Thorax Center Bad Nauheim Germany; ^2^ Department of Neurology University Hospital Giessen and Marburg Giessen Germany; ^3^ Cognitive Neuroscience at the Centre of Psychiatry University Giessen Giessen Germany; ^4^ Department of Psychology Justus‐Liebig University Giessen Germany; ^5^ Department of Psychocardiology Kerckhoff Heart and Thorax Center Bad Nauheim Germany; ^6^ Department of Cardiovascular Surgery University Hospital Giessen and Marburg Giessen Germany; ^7^ Department of Rehabilitation Kerckhoff Heart and Thorax Center Bad Nauheim Germany; ^8^ Department of Cardiac Surgery Kerckhoff Heart and Thorax Center Bad Nauheim Germany

**Keywords:** cardiac surgery, cognitive failures, cognitive training, health‐related quality of life, valve replacement

## Abstract

**Background:**

Heart surgery is a risk factor for objectively and subjectively assessable postoperative cognitive decline (POCD), which is relevant for everyday life. The aim of this study was to investigate whether early postoperative cognitive training has an impact on health‐related quality of life and cognitive failures in daily living after cardiac surgery.

**Methods:**

The study was a two‐arm, randomized, controlled, outcome‐blinded trial involving older patients undergoing elective heart valve surgery with extracorporeal circulation (ECC). Recruitment took place at the Departments of Cardiac Surgery of the Kerckhoff Clinic in Bad Nauheim (Germany) and the University Hospital in Giessen (Germany). The patients were randomized (1:1 ratio) to either a paper‐and‐pencil–based cognitive training group or a control group. We applied the Short Form Health Survey (SF‐36) and the Cognitive Failures Questionnaire (CFQ) prior to surgery and 3 months after the cognitive training. Data were analyzed in a per‐protocol fashion.

**Results:**

Three months after discharge from rehabilitation, the training group (*n* = 31) showed improvement in health‐related quality of life compared to the control group (*n* = 29), especially in role limitations due to emotional problems (*U* = −2.649, *p* = .008, *η* ^2^ = 0.121), energy and fatigue (*F*[2.55] = 5.72, *p* = .020, *η* ^2^ = 0.062), social functioning (*U* = −2.137, *p* = .033, *η* ^2^ = 0.076), the average of all SF‐36 factors (*U* = −2.374, *p* = .018, *η* ^2^ = 0.094), health change from the past year to the present time (*U* = −2.378, *p* = .017, *η* ^2^ = 0.094), and the mental component summary (*U* = −2.470, *p* = .013, *η* ^2^ = 0.102).

**Conclusion:**

As our cognitive training has shown beneficial effects, this intervention could be a promising method to enhance health‐related quality of life after cardiac surgery.

## Graphical abstract

The aim of this study was to investigate whether early postoperative cognitive training has an impact on health‐related quality of life and cognitive failures in daily living after cardiac surgery. The study was a 2‐arm, randomized, controlled, outcome‐blinded trial involving older patients undergoing elective heart valve surgery with extracorporeal circulation. As our cognitive training has shown beneficial effects, this intervention could be a promising method to enhance health‐related quality of life after cardiac surgery.

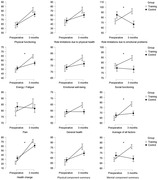

## 1 INTRODUCTION

Neurocognitive complications have been described following cardiac surgery. These include delirium (Kotfis et al., 2018) and postoperative cognitive decline (POCD) (Greaves et al., 2019), which both potentially lead to reduced quality of life (Newman et al., 2001) or increased mortality (Steinmetz et al., 2009). POCD often appears to be subclinical and therefore remains unrecognized. Nevertheless, patients and their relatives report a decrease in cognitive abilities in daily living up to at least 3 months after cardiac surgery (Kastaun et al., 2016). Taken together, subjectively assessed POCD and health‐related quality of life represent a clinically relevant intervention target. The pathogenesis of POCD involves several preoperative risk factors, such as depression (Kadoi et al., 2011; Tully et al., 2009), anxiety (Andrew et al., 2000; Tully et al., 2009) or mild cognitive impairment (Bekker et al., 2010). Furthermore, perioperative mechanisms are thought to cause and promote POCD. These include anesthesia, particularly cerebral (micro‐ and macro‐)embolization, and neuroinflammation (Berger et al., 2018). In addition, preoperative administration of dexamethasone ameliorates the inflammatory response provoked by surgery, which is related to a reduction in the incidence of POCD (Glumac et al., 2017). As we have recently demonstrated in a prospective, randomized controlled interventional study, early postoperative paper‐and‐pencil–based cognitive training can reduce the incidence of objectively measurable POCD (Butz et al., 2022). To further elucidate the clinical relevance of our cognitive training, this report evaluates the training‐induced effects on health‐related quality of life and cognitive failure in daily living after cardiac surgery. Our hypothesis is that “there is a difference between the control group and the training group in relation to health‐related quality of life and cognitive failures in daily living.”

## 2 METHODS

### 2.1 trial design and enrolment

This study was a bicentered, two‐arm, 1:1 randomized, controlled trial, conducted at following locations: The Department of Cardiac Surgery of the Kerckhoff Heart and Thorax Centre in Bad Nauheim, Germany; the Department of Cardiovascular Surgery at the University Hospital in Giessen, Germany; and the Department of Rehabilitation at the Kerckhoff Clinic in Bad Nauheim, Germany. The study, including informed consent, has been approved by the Ethics Committee of the Justus‐Liebig University Giessen (Ref.: 28/14), complies with the Declaration of Helsinki, and is registered with the German Clinical Trials Register (ID: DRKS00015512). The study protocol was published in advance (Butz et al., 2019).

The study coordinator screened the patient information on elective cardiac surgery for eligibility criteria. Potential participants received detailed information about the study project. Written informed consent was signed if the patient agreed.

Age, sex, education, body mass index, preexisting conditions, and the severity partition for left ventricular ejection fraction (Lang et al., 2015) were documented at baseline. Perioperatively, duration of surgery and extracorporeal circulation (ECC), cross‐clamp time, and invasive ventilation time were recorded. Postoperative complications, including delirium, were documented. After acute hospitalization, patients were directly transferred to the Department of Rehabilitation at the Kerckhoff Clinic in Bad Nauheim, Germany, where both groups received inpatient cardiac rehabilitation therapies that were individualized and based on the International Classification of Functioning. Key features of the therapeutic treatments were endurance and strength training, respiratory gymnastics, and educational lectures. Patients received 12–14 therapy sessions per week. In addition, patients of the training group underwent a multidomain cognitive intervention consisting of paper‐and‐pencil exercises that started about 1 week after surgery and lasted about 15 days until discharge from the rehabilitation clinic. A detailed description of the development and concept of the cognitive training (Butz et al., 2019) and its beneficial effects on cognition (Butz et al., 2022) have been published in advance.

### 2.2 Inclusion and exclusion criteria

Inclusion criteria included elective aortic or mitral valve replacement/reconstruction with or without coronary artery bypass crafting under ECC and sufficient knowledge of German. Exclusion criteria comprised history of stroke, psychiatric or neurological diseases, and health insurance that did not support postoperative rehabilitation at the Kerckhoff Clinic.

### 2.3 Randomization

A computer‐generated list with a 1:1 blocked allocation ratio was used for randomization. The randomization has randomly varied block sizes, and the study coordinator generated, sequentially numbered, and concealed it prior to the start of the study. After preoperative neuropsychological assessment, the study coordinator assigned the patients to the cognitive training group or the control group.

### 2.4 Blinding

Surgeons, neurologists, and neuropsychologists involved in the outcome variables were blinded for randomization status.

### 2.5 Outcome measures

The results of the primary outcome of the study have been published in advance (Butz et al., 2022). Secondary outcomes published in the present paper are the effect of cognitive training on health‐related quality of life and subjectively assessed cognitive failure in daily living at 3 months after the cognitive training.

### 2.6 Questionnaires

To reveal cognitive failures in daily living, we used the Cognitive Failures Questionnaire (CFQ), which is the most widely used instrument to assess self‐reported cognitive failures (Carrigan & Barkus, 2016). Study patients completed a validated German 25‐item version of the Cognitive Failures Questionnaire for self‐assessment (s‐CFQ) (Klumb, 1995). The patients’ close relatives responded to an 8‐item Cognitive Failures Questionnaire to evaluate foreign assessment (f‐CFQ) (Broadbent et al., 1982). Both have to be answered on a 5‐point scale from “never” to “very often.” The questionnaires examine the frequency of failures in daily living related to memory, attention, action, and perception. Because memory impairment is an important element that can affect every day functioning, the s‐CFQ was supplemented by 4 additional questions related to memory failures, taken from the validated German version of the Memory Complaint Questionnaire (MCQ) (Heß, 2005). We calculated various models to assess self‐reported cognitive failures. First, we averaged all items to a one‐factor value. Since several cognitive functions are integrated into the averaged one‐factor value and we did not overlook training effects on specific cognitive factors (e.g., distractibility, memory for names, misdirected actions), we analyzed several CFQ factor models that have already been described (Larson et al., 1997; Pollina et al., 1992; Rast et al., 2009; Wallace et al., 2002). According to the CFQ factor models, we calculated a single factor of the 4 memory questions taken from the MCQ.

We assessed health‐related quality of life using the 36‐Item Short Form Health Survey (SF‐36, Version 1.0) (Bullinger & Kirchberger, 1998). The SF‐36 includes 36 items covering 8 health‐related factors, including physical functioning (10 items), role limitations due to physical health (4 items), role limitations due to emotional problems (3 items), energy/fatigue (4 items), emotional well‐being (5 items), social functioning (2 items), pain (2 items), and general health (5 items). Furthermore, we determined a total score across all 8 factors, as well as a 2‐factor model, indicating the physical component summary (physical functioning, role limitations due to physical health, pain, general health) and mental component summary (role limitations due to emotional problems, energy/fatigue, emotional well‐being, and social functioning). The answers provided by the patients within the factors refer to the last 4 weeks, except for the factor physical functions and the first question of the factor general health, which refer to the present state of health. Furthermore, it also contains a single item (item 2, health change), which gives an indication of the extent to which the present health has changed in relation to the past year. The SF‐36 was scored using the RAND scoring method (Hays et al., 1993). Each item in the questionnaire was assigned a score from 0 to 100, with a higher score indicating a better health state.

For all questionnaires, the CFQ, the MCQ, and the SF‐36 handling with missing data were as follows. If the patients answered at least 50% of all items per factor, per time point, the mean score of this factor was calculated to determine the values of the factors. Items that were left blank (missing data) were not considered. Therefore, the factor values represent the average for all items of a factor that the respondent responded to.

Since worries about one`s cognition could have an impact on self‐reported cognitive failures in a way that depressed people answering themselves more conservative (Könen & Karbach, 2020; Wilhelm et al., 2010), we considered depression values as a control variable taken from the validated German version of the Hospital Anxiety and Depression Scale (HADS‐D) (Herrmann‐Lingen et al., 2011).

### 2.7 Statistical analyses

We carried out a sample size calculation for the primary outcome of our study (cognitive training–related effect on objectively assessed cognition), which was published in advance (Butz et al., 2022). Therefore, we did not perform any sample size calculation for this report, which refers to the secondary outcomes of our trial, and we analyzed the data exploratively.

To determine the effect of cognitive training on cognitive failure in daily living and health‐related quality of life, we conducted analyses of covariance (ANCOVAs) with the postoperative questionnaire value as the dependent variable, groups (control group/training group) as the fixed factor, and the preoperative questionnaire value as the covariate. We tested assumptions for ANCOVAs using the Levene test (homogeneity of variance between groups) for the dependent variable and a statistically significant correlation between the dependent variable and covariate (preoperative test value) calculated using the Pearson product‐moment correlation. As a further assumption, we checked the distribution and Q‐Q plots for normality. When assumptions for ANCOVA were violated, we calculated difference values between pre‐ and posttests, followed by the Mann–Whitney *U*‐test for between‐subject effects. To control for the possibility of confounder variables that could affect the results, we conducted correlation analysis between potentially confounding variables (e.g., continuous demographic variables, perioperative details, changes in anxiety and depression) and changes between pre‐ and postoperative SF‐36 and CFQ assessment. In the case of these variables’ significant contributions, we implemented them additionally to the preoperative values as further covariates to the ANCOVA. We checked all data entries for inconsistent values.

Subjective‐POCD was defined as a decline and subjective‐POCI as an improvement from pre‐ to postassessment of at least 1 SD (Kastaun et al., 2016) in all considered CFQ models. To measure the difference of 1 SD between pre‐ and postassessment, we used Z‐scores, which were calculated by the difference of the individual raw values from the mean value of the total baseline data divided by the SD of the total baseline data. To reveal the training effect on cognition, frequencies of dichotomous (yes/no) subjective‐POCD and subjective‐POCI variables were compared with Pearson's χ^2^ test between the groups.

We give the effect size as *η* ^2^ and set the criterion for statistical significance at *p* < .05. We evaluated our data set with a per‐protocol analysis, and we performed all analyses using the statistical software SPSS (version 22) and JASP (version 0.12.2).

## 3 RESULTS

Between July 13, 2016 and January 8, 2020, a total of 130 patients were enrolled, randomized, and tested preoperatively. The last patient was tested on February 27, 2020 for the 3‐month follow‐up. The recruitment has concluded.

**FIGURE 1:**
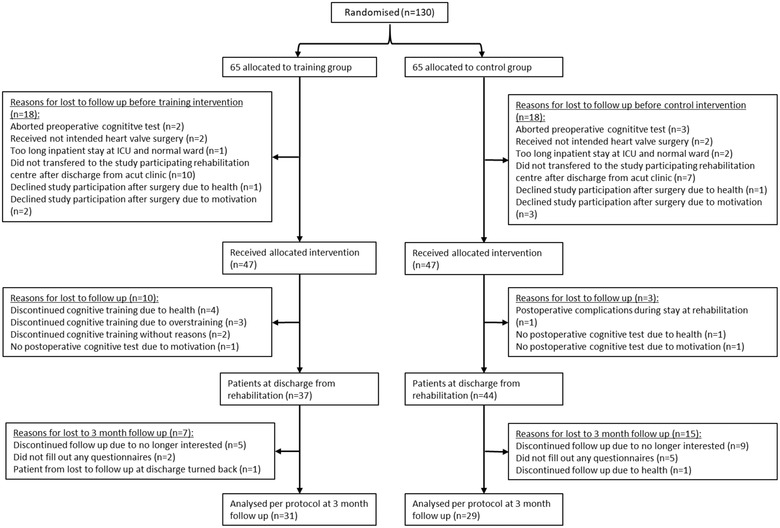
Consolidated Standards of Reporting Trials (CONSORT) flow chart illustrating all steps in the study from randomization to follow‐up and analysis. ICU = intensive care unit.

After randomization of 130 patients, 36 (training group *n* = 18, control group *n* = 18) were lost to follow‐up before the training or control intervention had begun. Thus, 94 patients (training group *n* = 47, control group *n* = 47) were considered for the baseline sample. Table 1 lists the baseline characteristics. No statistical significant group differences have been shown in the baseline data. Thirteen patients (training group *n* = 10, control group *n* = 3) were lost to follow‐up, whereas 81 patients (training group *n* = 37, control group *n* = 44) remained at discharge from rehabilitation. Thus, about 80% of the patients completed the training. Another 21 patients (training group *n* = 6, control group *n* = 15) were lost to the 3‐month follow‐up, resulting in 60 patients (training group *n* = 31, control group *n* = 29) for analysis. Five patients (training group *n* = 2, control group *n* = 3) did not answer enough questions within the questionnaires to complete the missing data, resulting in different sample sizes in the statistical tests (see Table 2). Figure 1 outlines the reasons that patients were lost to follow‐up. The training intervention lasted 14.86 (SD = 2.507) days and did not cause any adverse events.

**TABLE 1:** Baseline demographics and characteristics of the intention to treat the population.

	Training (*n* = 47)	Control (*n* = 47)
Demographics		
Age (years)	71.2 (4.7)	73.0 (4.9)
Sex		
Women	8 (17%)	13 (28%)
Men	39 (83%)	34 (72%)
Education (years)	13.5 (3.0)	13.4 (3.0)
Medical history		
Body mass index (kg/m^2^)	27.7 (3.8)	26.6 (3.8)
Arterial hypertension	31 (66%)	31 (66%)
Diabetes mellitus	10 (21%)	5 (11%)
Renal insufficiency	4 (9%)	7 (15%)
Dyslipidemia	39 (83%)	37 (79%)
LV EF (mildly abnormal)	5 (11%)	6 (13%)
LV EF (moderately abnormal)	3 (6%)	1 (2%)
Heart failure	10 (21.3%)	9 (19.1%)
Type of surgery		
AVR	23 (50%)	22 (47%)
AVR+CABG	18 (38%)	19 (40%)
MVR	4 (9%)	1 (2%)
MVR+CABG	0 (0%)	3 (6%)
AVR+MVR	2 (4%)	2 (4%)
Perioperative details		
Duration of surgery (minutes)	190.3 (38.1)	200.6 (58.9)
Duration of extracorporeal circulation (minutes)	96.5 (26.5)	105.2 (37.5)
Cross‐clamp time (minutes)	69.1 (19.9)	74.7 (26.7)
Ventilation time invasive (minutes)	616.2 (331.3)	588.1 (213.6)
Postoperative complications		
Delirium	2 (4%)	4 (9%)
Arrhythmia	18 (38%)	21 (45%)
Atrial fibrillation	18 (38%)	17 (36%)
Renal insufficiency	6 (13%)	6 (13%)
Acute blood loss anemia	10 (21%)	12 (26%)
Transient ischemic attack	1 (2%)	0 (0%)
Dysathria/aphasia	0 (0%)	1 (2%)
Medical details at admission to rehabilitation		
Blood pressure (systolic; mmHG)	128.1 (12.7)	127.7 (18.4)
Blood pressure (diastolic; mmHG)	74.5 (11.1)	71.4 (10.6)

*Note*: Data include means (SD) or number of subjects (%). LF EV = left ventricular ejection fraction, defined according to Lang et al. (2015). AVR = aortic valve replacement. CABG = coronary artery bypass grafting. MVR = mitral valve replacement/reconstruction. Renal insufficiency was defined by a creatinine value above the in‐house norms (men: > 1.2 mg/dL, women: > 0.9 mg/dL).

**TABLE 2:** SF‐36 results per group and assessment time for the per‐protocol analysis.

SF‐36 Factor	Timepoint	Group	Mean	SD	*n*	*p* Value	*η* ^2^
Physical functioning	Baseline	Control	64.563	22.055	28		
		Training	63.553	24.420	31		
	3‐month follow‐up	Control	76.190	22.394	28		
		Training	80.932	14.156	31		
						.568	0.006
Role limitations due to physical health	Baseline	Control	48.077	42.381	26		
		Training	43.011	41.337	31		
	3‐month follow‐up	Control	60.577	42.528	26		
		Training	71.774	35.790	31		
						.126	0.041
Role limitations due to emotional problems	Baseline	Control	85.057	31.605	29		
		Training	72.414	37.869	29		
	3‐month follow‐up	Control	66.667	43.644	29		
		Training	91.954	21.185	29		
						.008	0.121
Energy/fatigue*	Baseline	Control	51.296	16.675	27		
		Training	48.548	16.842	31		
	3‐month follow‐up	Control	56.975	21.593	27		
		Training	64.570	15.623	31		
						.020	0.062
Emotional well‐being	Baseline	Control	73.778	14.606	27		
		Training	72.710	18.927	31		
	3‐month follow‐up	Control	76.556	17.579	27		
		Training	81.290	12.623	31		
						.462	0.009
Social functioning	Baseline	Control	79.741	21.499	29		
		Training	81.855	16.085	31		
	3‐month follow‐up	Control	78.448	25.638	29		
		Training	89.516	17.410	31		
						.033	0.076
Pain	Baseline	Control	83.276	19.017	29		
		Training	79.274	26.459	31		
	3‐month follow‐up	Control	81.810	22.018	29		
		Training	86.129	19.936	31		
						.336	0.016
General health*	Baseline	Control	59.226	13.174	28		
		Training	57.056	15.175	31		
	3‐month follow‐up	Control	59.152	14.900	28		
		Training	64.516	18.989	31		
						.067	0.04
Average of all factors	Baseline	Control	67.648	13.699	29		
		Training	64.611	16.514	31		
	3‐month follow‐up	Control	69.515	20.672	29		
		Training	78.497	14.049	31		
						.018	0.094
Health change (Item 2)	Baseline	Control	32.759	21.238	29		
		Training	22.581	14.937	31		
	3‐month follow‐up	Control	61.207	28.020	29		
		Training	71.774	27.189	31		
						.017	0.094
Physical component summary	Baseline	Control	63.020	18.257	29		
		Training	60.724	19.413	31		
	3‐month follow‐up	Control	69.547	20.666	29		
		Training	75.838	18.003	31		
						.173	0.031
Mental component summary	Baseline	Control	72.517	16.029	29		
		Training	68.647	17.245	31		
	3‐month follow‐up	Control	69.273	23.630	29		
		Training	81.156	11.352	31		
						.013	0.102

*Note*: All test parameters were calculated with difference values between pre‐ and posttests, followed by Mann–Whitney *U*‐test for between‐subject effects, except those marked with an asterisk (*), which were calculated by ANCOVA. Data include means (SD). *n* = number of cases. *η* ^2^ = effect size

**FIGURE 2:**
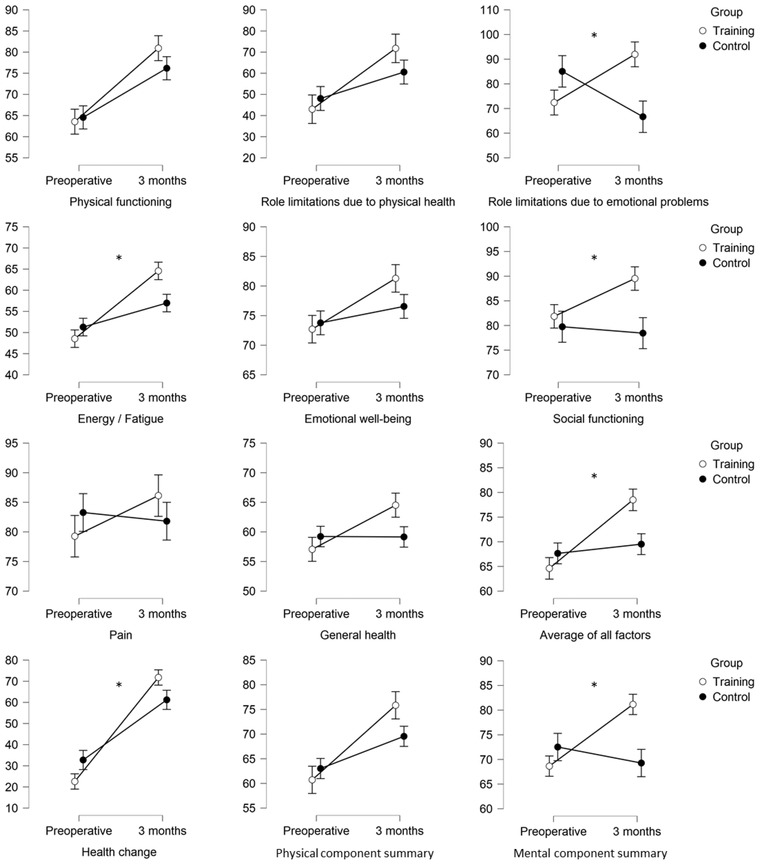
Interaction effects of all SF‐36 factors between the training group and control group. Shown are the mean values (higher scores indicating a better health state), including SE bars for preoperative testing and 3 months after discharge from the rehabilitation clinic. Statistical significant interaction effects with a *p* value of <.05 are marked with an asterisk (*).

Three months after discharge from rehabilitation, some improvements in health‐related quality of life were evident for the training group compared to the control group. These improvements have been seen in role limitations due to emotional problems (*U* = −2.649, *p* = .008, *η* ^2^ = 0.121), energy and fatigue (*F*[2.55] = 5.72, *p* = .020, *η* ^2^ = 0.062), social functioning (*U* = −2.137, *p* = .033, *η* ^2^ = 0.076), the average of all SF‐36 factors (*U* = −2.374, *p* = .018, *η* ^2^ = 0.094), health change from the past year to the present time (*U* = −2.378, *p* = .017, *η* ^2^ = 0.094), and the mental component summary (*U* = −2.470, *p* = .013, *η* ^2^ = 0.102). Figure 2 shows the interaction effects. Table 2 provides a full description of the results of all SF‐36 factors.

In the adjusted ANCOVA with potentially confounding variables, cross‐clamp time (*F*[3.54] = 6, *p* = .018, *η* ^2^ = 0.058) and changes in depression over time (*F*[3.54] = 4.19, *p* = .046, *η* ^2^ = 0.046) contributed significantly to the SF‐36 factor energy/fatigue.

There were no statistically significant or clinically relevant interaction effects between the control group and the training group in all considered s‐CFQ models, f‐CFQ, and MCQ.

## 4 DISCUSSION

Our key findings were beneficial effects 3 months after discharge from rehabilitation in several health‐related quality‐of‐life domains. These have been found in role limitations due to emotional problems, energy and fatigue, social functioning, the average of all SF‐36 factors, health change from the past year to the present time, and the mental component summary. The treatment success of cardiac surgery is potentially based on the objective clinical or physiological status. The subjective patient‐centered changes (physical and psychological) seem to be important as well and may especially contribute to the treated patient's quality of life (Koch et al, 2008). Therefore, we used the SF‐36 questionnaire to assess postoperative patient's physical and mental processes in the context of a cognitive training program.

A computerized approach has shown enhancements for health‐related quality of life after postoperative cognitive training, which is in line with our results (Ajtahed et al., 2019). In addition, healthy older people showed various improvements in quality‐of‐life parameters (e.g., role limitations due to emotional problems, social functioning, role limitations due to functional limitations) after a controlled cognitive intervention with positive posttraining effects at a 3‐month follow‐up (Shati et al., 2021). Cognitive training–related improvements of depressive symptoms also exist in older adults with subclinical cognitive decline (Gooding et al., 2016). Reduced postoperative health‐related quality of life can increase mortality after cardiac surgery (Steinmetz et al., 2009), making the beneficial effects of cognitive training particularly important.

Cognitive abilities were evaluated as an additional outcome in this study because they contribute substantially to independence, personality, and self‐image for elderly patients, and also represent another important factor of health‐related quality of life. In the present investigation, the subjective assessment of cognitive failures in everyday living has not shown a difference between the groups. Therefore, we assumed that the CFQ‐questionnaire might not be sensitive enough to reveal alterations from the cognitive training. There is evidence that subjective assessments of cognitive ratings are unrelated to objective testing of cognitive performance (Brück et al., 2019; Carrigan & Barkus, 2016). Studies have discussed whether ICU survivors or elderly people, who may be more likely to be affected by cognitive impairment, inadequately estimate their own cognitive performance. This could lead to a lack of correlation with objective tests (Brück et al., 2019). In addition, it is possible that people underestimate cognitive deficits in the daily life of relatives who has survived a potentially life‐threatening disease or medically necessary surgery.

Nevertheless, we have shown that cognitive training is associated with psychometrically confirmed improvements in postoperative cognition (Butz et al., 2022). We assume that this effect can be transferred to psychologically relevant everyday situations and, therefore, increase health‐related quality of life.

A few limitations should be mentioned. First, a comparison with patients without the use of an ECC or a healthy control cohort does not seem possible, as we only examined patients who had been operated with ECC. Related to the results reported here and a previously published article concerning the objectively determined frequency of POCD (Butz et al., 2022), another limitation of our study emerges. We did not evaluate the incidence of “postoperative mild and major neurocognitive disorders” as defined by Evered et al. (2018), which is recommended for the research of POCD. This was not possible because some of the following research criteria were not implemented in our study protocol: the questions about postoperative alterations in activity of daily living (ADL) and the patient´s subjective decrease of postoperative cognition, specifically referring to heart surgery. Furthermore, the use of a healthy control group to calculate a reliable change index (controls for time and practice effects), which is also recommended to calculate POCD (Rasmussen et al., 2001), was not involved in our study. Furthermore, we did not evaluate if and how the patients practiced some cognitive‐enhancing activities (e.g., playing games, reading books, pronounced social activities) between the end of cognitive training and the 3‐month follow‐up, which could have also had an impact on cognitive plasticity (Xu et al., 2019). Since patients in the cognitive training group learned specifically what type of training material could be used for cognitive improvement, they would be more likely to come up with the idea of using similar material in the postrehabilitation period than the control group, who were only made aware of the potential of cognitive‐enhancing training through the consent form and information sheet. Compared to the control group, the training group showed shorter duration of surgery, extracorporeal circulation, and cross‐clamp time. As duration of surgery and anesthesia are reported to be risk factors for POCD (Vu & Smith, 2022), this could also affect health‐related quality of life (HQL), as an association between POCD and HQL has been found (Phillips‐Bute et al., 2006). However, the duration of surgery, extracorporeal circulation, and cross‐clamp time seems unassociated with HQL (Sanders et al., 2022). In addition, no significant group differences were shown for these factors in our sample, and a post hoc ANCOVA with these factors as control variables showed no changes in between‐group effects from statistically significant to nonsignificant results. We counted the incidence of POD using medical records. As we did not perform a standardized daily assessment of POD in the ICU and normal ward, the frequency of POD cases in our sample may be underreported. Because health insurance for patients transferred from the acute clinic to the rehabilitation center only covers patients of retirement age, the results of cognitive training are limited to this particular cohort.

Since our training concept was able to decrease POCD or maintain and improve health‐related quality of life after cardiac surgery, it could also be useful for noncardiac surgery patients potentially affected by POCD. In general, it may also be beneficial for patients suffering cognitive impairment after stroke or in the context of dementia. In addition, our concept is designed to be continued in an ambulatory setting after clinical implementation (e.g., in a home‐based environment) or performed in a home‐based setting prior to surgery that has the potential to impair cognition. Preoperative cognitive training might build up a so‐called cognitive reserve, which could provide prophylactic protection of the brain (Saleh et al., 2015).

## AUTHOR CONTRIBUTIONS

Marius Butz: Conceptualization; data curation; formal analysis; funding acquisition; investigation; methodology; project administration; supervision; validation; visualization; writing‐original draft. Tibo Gerriets: Conceptualization; funding acquisition; methodology; project administration; supervision; writing–review & editing. Gebhard Sammer: Conceptualization; methodology; project administration; supervision; validation; writing–review & editing. Jasmin El‐Shazly: Conceptualization; funding acquisition; investigation; methodology; project administration; supervision; validation; writing–review & editing. Marlene Tschernatsch: Writing–review & editing. Patrick Schramm: Writing–review & editing. Thorsten R. Doeppner: Writing–review & editing. Tobias Braun: Writing–review & editing. Andreas Boening: Resources; writing–review & editing. Thomas Mengden: Conceptualization; resources; writing–review & editing. Yeong‐Hoon Choi: Writing–review & editing. Markus Schoenburg: Conceptualization; funding acquisition; methodology; project administration; resources; supervision; writing–review & editing. Martin Juenemann: Conceptualization; funding acquisition; methodology; project administration; supervision; writing–review & editing.

## CONFLICT OF INTEREST STATEMENT

The authors declare that they have no conflict of interests.

### PEER REVIEW

The peer review history for this article is available at https://publons.com/publon/10.1002/brb3.2915.

## Data Availability

Deidentified participants’ data analyzed during the current study are available from the corresponding author on reasonable request.
